# Revealing the Hidden Mechanism of Enhanced Responsivity of Doped p-i-n Perovskite Photodiodes via Coupled Opto-Electronic Model

**DOI:** 10.3390/molecules27196223

**Published:** 2022-09-22

**Authors:** Dan Wu, Hechun Zhang, Haochen Liu, Wenhui Li, Xiangtian Xiao, Kanming Shi, Taikang Ye, Jiayun Sun, Zhaowen Lin, Jing Liu, Mingxia Qiu, Aung Ko Ko Kyaw, Kai Wang

**Affiliations:** 1 College of New Materials and New Energies, Shenzhen Technology University, Shenzhen 518118, China; 2 Department of Electrical and Electronic Engineering, Southern University of Science and Technology, Shenzhen 518055, China

**Keywords:** perovskite photodetector, coupled opto-electronic model, p-i-n photodiode

## Abstract

Organic-inorganic halide perovskites have demonstrated preeminent optoelectronic performance in recent years due to their unique material properties, and have shown great potential in the field of photodetectors. In this study, a coupled opto-electronic model is constructed to reveal the hidden mechanism of enhancing the performance of perovskite photodetectors that are suitable for both inverted and regular structure doped p-i-n perovskite photodiodes. Upon illumination, the generation rate of photogenerated carriers is calculated followed by carrier density distribution, which serves as a coupled joint to further analyze the recombination rate, electric field strength, and current density of carriers under different doping types and densities. Moreover, experiments were carried out in which the doping types and densities of the active layer were regulated by changing the precursor ratios. With optimal doping conditions, the inverted and regular perovskite photodiodes achieved an external quantum efficiency of 74.83% and 73.36%, and a responsivity of 0.417 and 0.404 A/W, respectively. The constructed coupled opto-electronic model reveals the hidden mechanism and along with the doping strategy, this study provides important guidance for further analysis and improvement of perovskite-based photodiodes.

## 1. Introduction

Photodetectors are vital for a wide variety of industrial and scientific applications, including national defense, image sensing, optical communication, climate monitoring, medical treatment, etc. [1,2,3,4,5,6]. Nowadays, commercialized photodetectors are generally made of inorganic semiconductor materials, such as GaN, Si, and InGaAs, for various detection spectra. However, these commercialized photodetectors usually suffer from process complexity, expensive cost, and mechanical inflexibility, which have gradually become the main drawbacks that limit their broader applications [7,8]. In recent years, the emergence of perovskite materials has injected new vitality into the photodetectors owing to their outstanding optoelectronic properties including the tunable bandgap, long carrier diffusion length, high absorption coefficients, and so on [9,10,11].

Typically, there are two types of perovskite photodetectors architectures, i.e., lateral and vertical structures. Photodetectors, mainly the photodiodes, with vertical structures have smaller electrode spacing accompanied by shorter carrier transport lengths than their counterparts with lateral structures. Therefore, the photodiodes generally demonstrate a faster response speed with a lower driving bias [12,13,14]. Photodiodes usually bear a sandwich structure, where the photoactive layer is sandwiched between an electron transport layer (ETL) and a hole transport layer (HTL). To enhance the performance of the photodiodes, lots of reported strategies focus on material modifications to reduce the trap density, increase carrier lifetime, and enhance carrier mobility [15,16]. For example, Luo and co-workers fabricated MAPbBr_3_ single crystal p-n perovskite homojunction photodiode through the controlled incorporation of Bi^3+^ ions in solution, which demonstrated a high responsivity of 0.62 A/W [17]. Furthermore, Li et al. proposed a self-powered photodetector based on perovskite/CdS with a high responsivity of 0.48 A/W by bending the gradient band to promote the photogenerated carrier transfer and hinder the recombination at the interface [18]. However, most of the material modification strategies could only address specific perovskite materials or certain perovskite/transport layer combinations. On the other hand, device engineers have focused on the optimization of the structures of the device to improve device performance. Our group successfully modulated the unintentional doping of the active layer to construct the interfacial built-in electric field at the perovskite/transport layer interface far from the incident light side and, thus, obtained high responsivity of the photodetectors [19]. Fang et al. demonstrated self-powered photodetectors with a novel p-p heterojunction, which generated a built-in electric field to separate electron-hole pairs to benefit device performance [20]. These strategies can be suitable for a variety of material combinations regardless of inverted or regular structure photodiodes. 

To further enhance the performance of perovskite photodiodes, an in-depth analysis of device physics is critically important. Previous studies have constructed optical models to analyze current, intensity, and absorption [21,22,23]. In addition, some electrical models are established to investigate the current-voltage characteristics and carrier transport of photodetectors [24,25,26]. Usually, the reported works consider the light absorption (as the optical model) and the carrier transportation (as the electrical model) separately where the interrelated connection between the two are frequently neglected. However, this coupled opto-electronic model is the core part of a comprehensive and thorough analysis of the generation rate, recombination rate, and carrier density under the modulation of active layer doping in perovskite photodiodes followed by a detailed discussion about the physical quantities such as the external quantum efficiency and responsivity of devices. A clear analysis of the physical schema in the perovskite photodiode is still unclear, specifically for devices with built-in electric field modulation, which must be elucidated explicitly to further enhance the performance of the perovskite photodiodes.

In this work, a coupled opto-electronic model is constructed to thoroughly analyze the influence of the built-in electric fields for the doped p-i-n perovskite photodiode with both regular and inverted structures along with experimental validation by a rational perovskite doping strategy. Upon illumination, the generation rate of photogenerated carriers is calculated followed by carrier density distribution, which serves as a coupled joint to further analyze the recombination rate, electric field strength, and current density of the carriers. Experimentally, the doping type and density of the perovskite were regulated by changing the proportion of precursor, and various characterizations were performed to confirm the establishment of a built-in electric field. Both the coupled opto-electronic model and the experiments prove the positive influence of the built-in electric field on the responsivity enhancement of the perovskite photodiodes, which provides important guidance for future design and improvement of perovskite photodiodes.

## 2. Results and Analysis

### 2.1. Analysis of the Built-in Electric Fields by the Coupled Opto-Electronic Model

**Figure 1:**
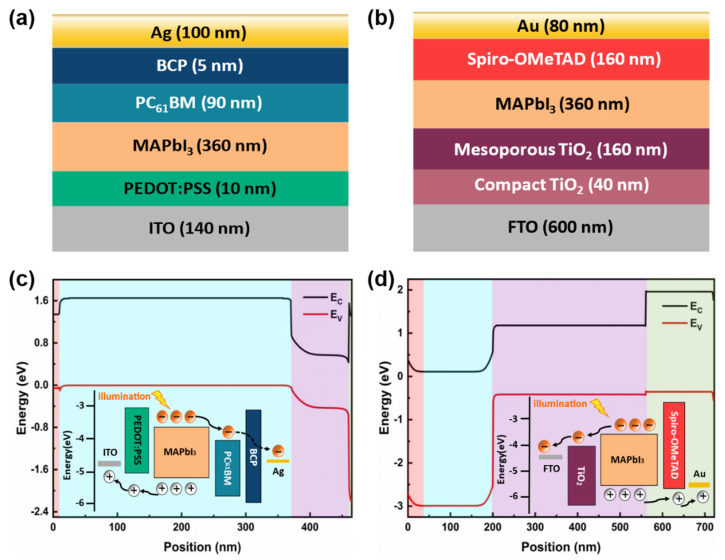
The structural schematic of both (**a**) inverted and (**b**) regular perovskite photodiodes and the diagrams of energy bands and the process of carrier generation, separation, and transport of (**c**) the inverted and (**d**) regular perovskite photodiodes.

Figure 1 depicts the energy band diagrams of the inverted and regular structure photodiodes and the process of carrier generation, separation, and transport. Without considering the doping type and density of the perovskite, the carrier extractions at the interfaces are only influenced by the highest occupied molecular orbital (HOMO)/lowest unoccupied molecular orbital (LUMO), the Fermi levels, and the bandgaps of the respective functional layers. Due to the band alignment, the electrons and holes drift in opposite directions. The electrons are injected into the ETL and transported to the cathode, whereas the holes are injected into the HTL and transported to the anode. The extraction of photo-induced electrons and holes occurs at the perovskite/ETL and perovskite/HTL interfaces, respectively [27,28,29]. To analyze the performance of the photodiodes with doped active layer, a coupled opto-electronic model is created for inverted (Indium Tin Oxide (ITO)/Poly (3,4-Ethylenedioxythiophene):Poly (Styrenesulfonate) (PEDOT:PSS)/Methylammonium Lead Iodide (MAPbI_3_)/(6,6) Phenyl-C_61_-Butyric Acid Methyl Ester (PC_61_BM)/Bathocuproine (BCP)/Ag) and regular (Fluorine-doped Tin Oxide (FTO)/Compact Titanium Dioxide (TiO_2_)/Mesoporous TiO_2_/MAPbI_3_/2,2′,7,7′-Tetrakis (N,N-di (4-Methoxyphenyl)Amino)-9,9′-Spirobifluorene (Spiro-OMeTAD)/Au) structures. The schematics of both inverted and regular structure photodiodes are illustrated in Figure 1a,b, where the thickness of each layer is indicated on the respective images.

**Figure 2:**
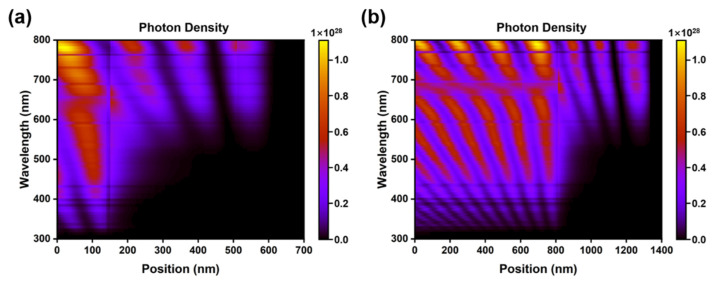
Simulated the photon density under AM 1.5 G illumination by GPVDM, where (**a**) are for the inverted-structure photodiode. Inset: Generation rates of photogenerated carriers at different wavelengths across perovskite active layer (**b**) are for the regular-structure photodiode.

To analyze carrier generation in perovskite photodiodes, the generation rates are calculated separately at five wavelengths ranging from 300 to 700 nm. As displayed in Figure 2, under light illumination, the perovskite layers absorb incident photons, and, due to the planar surface at each side of the active layer, resonances appear. It can be observed that the carrier generation at different wavelengths decays exponentially with the position coordinate, and the generation rate is maximum at the initial position, which is the position of light incidence. With the increase in incident wavelength, the absorption coefficient of perovskite and, correspondingly, the photon flux decreases gradually, which leads to a decrease in the carrier generation rate. The optical simulation software of GPVDM is used to simulate the photon spectral absorption at different thicknesses of devices under AM1.5G solar spectrum, which is illustrated in Figure 2a,b. The cut-off wavelength of the perovskite materials is 775.5nm, which can be estimated from the expression $E_{g}=\frac{hc}{\lambda }$, where $E_{g}$ (=1.6 eV) is the band gap of MAPbI_3_, h (=4.136 × 10^−15^ eV·s) is Planck’s constant, and c (=3 × 10^8^ m/s) is the light speed. By sectioning the photon density image parallel to the position of the active layer, the generation rate curves at different wavelengths are obtained.

**Figure 3:**
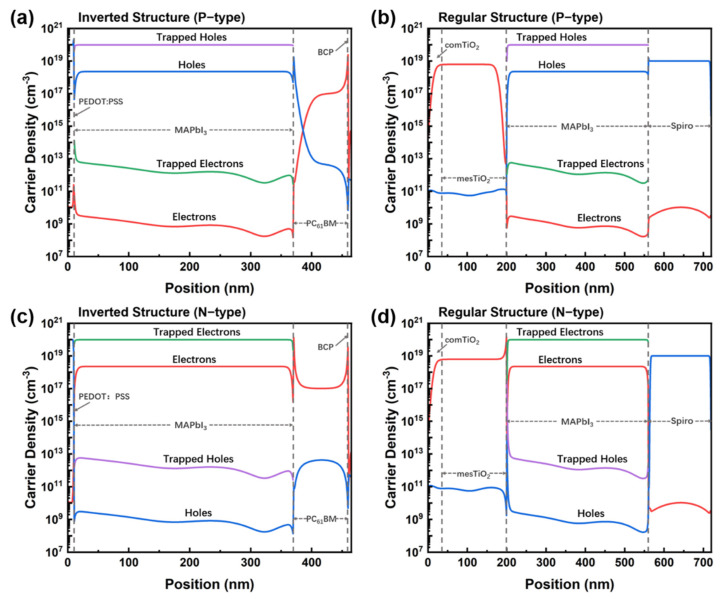
Carrier distribution under (**a**,**b**) P-doped and (**c**,**d**) N-doped perovskite conditions where (**a**,**c**) are for the inverted-structure photodiode and (**b**,**d**) for the regular-structure photodiode. The position refers to the cross-sections of the inverted structure and the regular structure, respectively. The dashed grey lines are used to identify each layer in the perovskite photodiodes.

Upon photocarrier generation, they are affected by factors such as electric field, recombination, and motion, and they exhibit spatial distribution. In the case of considering only 1D, Equations for calculating the carrier densities can be organized into the following nonlinear partial differential equations and the physical processes of carrier drift-diffusion, recombination, and generation are included in the two equations (Equations (1) and (2)). The carrier density distribution across the whole device structure is shown in Figure 3, where the majority and the minority of the carriers are discernable clearly in the P-doped and N-doped perovskite layers. The dashed blue lines are used to identify the layers in the perovskite photodiodes. It is shown that the density of the electrons and holes is changed nearby the perovskite/transport layer interface, demonstrating the role of the built-in E field’s influence in oriented carrier transport.

(1) $$-\mu_{n}\left(\frac{dn}{dx}\cdot \frac{dE}{dx}+n\cdot \frac{d^{2}E}{dx^{2}}\right)-\mu_{n}\frac{kT}{q}\frac{d^{2}n}{dx^{2}}-C_{t}N_{t}\frac{np-n_{i}^{2}}{\left(n+n_{t})+(p+p_{t}\right)}+G=0$$

(2) $$\mu_{p}\left(\frac{dp}{dx}\cdot \frac{dE}{dx}+p\cdot \frac{d^{2}E}{dx^{2}}\right)-\mu_{p}\frac{kT}{q}\frac{d^{2}p}{dx^{2}}-C_{t}N_{t}\frac{np-n_{i}^{2}}{\left(n+n_{t})+(p+p_{t}\right)}+G=0$$

**Figure 4:**
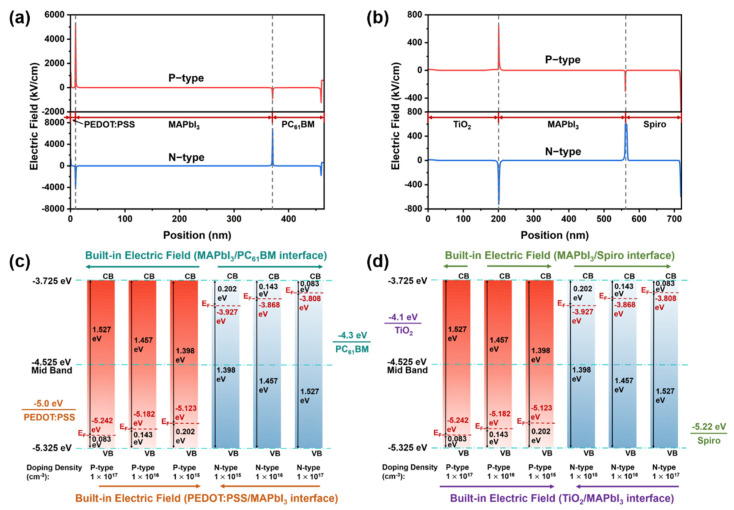
Built-in electric field distribution under P-doped and N-doped in (**a**) inverted and (**b**) regular photodiodes. The dashed grey lines are used to identify the layers in the devices from (**a**) to (**b**). Images of the interfacial *E* strength at (**c**) PEDOT:PSS/MAPbI_3_ and MAPbI_3_/PC_61_BM interfaces, (**d**) TiO_2_/MAPbI_3_ and MAPbI_3_/Spiro interfaces under different doping densities.

To further derive the carrier distribution of the interfacial electric field of P-doped and N-doped perovskite photodiodes with various structures, these physical processes were simulated using commercial software of Setfos 5.0, where the doping density is 1 × 10^20^ cm^−3^ for both P-type and N-type perovskite. Based on the carrier density, the electric field distribution of the inverted structure and regular structure devices can be calculated for P-doped and N-doped perovskite photodiodes, respectively. As shown in Figure 4a,b where the doping density is 1 × 10^20^ cm^−3^, there are two interfacial electric fields on either side of the perovskite layer. For example, in the inverted structure photodiodes, if the perovskite is doped with P-type, there is a PP^+^ heterojunction and a PN heterojunction formed at the interface of PEDOT:PSS/MAPbI_3_ and MAPbI_3_/PC_61_BM, respectively. Therefore, there is an *E* at the PEDOT:PSS/MAPbI_3_ interface pointing from MAPbI_3_ to the PEDOT:PSS, whereas there is another small *E* pointing in the opposite direction at the MAPbI_3_/PC_61_BM interface.

To further verify the effect of perovskite doping density and type on the built-in electric field, perovskite photodiodes with both inverted and regular structures were simulated, which are widely used in various types of optical and electrical performance simulation. The simulated structure and thickness of each layer of the perovskite photodiode are mentioned in the structural diagram of Figure 1a,b. The parameters used for the coupled opto-electrical model are listed in Tables S1 and S2, Supplementary Materials. At the interface between the perovskite active layers and the carrier transport layers, the interface electric fields are constructed due to the differences in energy levels. With the change in perovskite doping types and densities, the strength and direction of the interface electric fields change accordingly, as shown in Figure S2a,b, Supplementary Materials. To further reveal the reason for the electric field change, the shift in the Fermi level position of perovskite under different doping types and densities needs to be accurately dissected according to Figure 4c,d. In the case of perovskite P-doping, it is clearly observed that the Fermi level gradually approaches the valence band with increasing doping densities. In contrast, the Fermi level shifts to the CB with increasing doping densities under perovskite N doping. Due to the difference in energy levels of different materials, electrons flow from high energy levels to low energy levels, forming electric fields with different directions.

### 2.2. Doping Type and Density Changing of Perovskite by Precursor Ratio Variation

**Figure 5:**
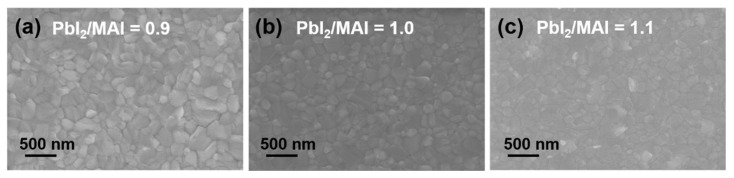
Scanning electron microscope (SEM) images. Surface SEM of MAPbI_3_ films on Glass/ITO/PEDOT:PSS with different PbI_2_/MAI ratio of (**a**) 0.9, (**b**) 1.0, (**c**) 1.1. The scale bars are 500 nm for all images.

**Figure 6:**
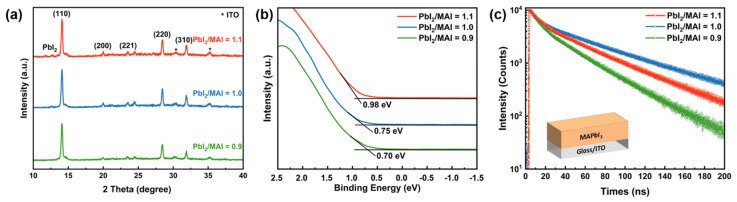
(**a**) X-ray diffraction (XRD) results of perovskite samples with various precursor ratios. “*” refers to ITO. (**b**) X-ray photoelectron spectroscopy (XPS) of MAPbI_3_ films under different precursor ratios. (**c**) Time-resolved photoluminescence (TRPL) measurements of the Glass/ITO/MAPbI_3_ sample under different precursor ratios.

Perovskite thin films were prepared by one-step solution-process deposition using lead iodide (PbI_2_) and methylammonium iodide (MAI) solutions. The specific preparation of the perovskite layer can be referred to in our previous work [19]. Unlike our previous work, the doping type and density of perovskite were controlled by changing the precursor ratio (PbI_2_/MAI), while the annealing temperature was fixed at 80 °C and the annealing time was 10 min. Figure 5 shows the scanning electron microscope (SEM) images of the MAPbI_3_ perovskite thin film morphology under different PbI_2_/MAI ratios. The films exert high-quality polycrystalline nature and have no discernible pinholes. It is worth noting that the grain size continuously decreased as the precursor ratio increased from 0.9 to 1.1 when the annealing temperature and time were fixed. More white flakes appeared in the films fabricated at a precursor ratio of 1.1, confirmed to be PbI_2_ by the X-ray diffraction (XRD) patterns. As shown in Figure 6a, it is observed that the perovskite films with different precursor ratios all exhibit similar crystal orientations and multiple diffraction peaks at 2θ values of 14.1°, 20.1°, 23.5°, 28.4°, and 31.9°, corresponding to the (110), (200), (221), (220), and (310) planes of MAPbI_3_, respectively. In addition, under different precursor ratios, the perovskite films all show strong (110) diffraction peaks and high crystallinity, which is beneficial to the transport of charge carriers in the perovskite active layers.

To further explore the doping properties of perovskite films, X-ray photoelectron spectroscopy (XPS) measurements were performed on the perovskite films prepared on Glass/ITO/PEDOT:PSS substrates. Figure 6b reveals that the Fermi level of perovskite gradually increases as the precursor ratios rise, which is far from the VB. Because perovskite has a band gap of 1.6 eV, it can be determined that with the increase in the precursor ratio, the perovskite changes from P-type doping to intrinsic, and then from intrinsic to N-type doping. When the precursor ratio is 0.9, the Fermi level is located at 0.70 eV from the VB, which indicates a P-type. As the precursor ratio increases, the Fermi level approaches the CB. For samples with a precursor ratio of 1.1, the Fermi level rises to 0.62 eV below the CB, manifesting as an N-type. Moreover, the elemental ratios of iodine and lead at the atomic ratios of different XPS elements are summarized and listed in Table S3, Supplementary Materials, to support the variation in doping type due to the change in the precursor ratio.

To investigate the quality of perovskite films under different precursor ratios, time-resolved photoluminescence (TRPL) measurement was used to test samples of Glass/ITO/MAPbI_3_, as observed in Figure 6c. In the TRPL spectrum, the photogenerated carriers recombine through dopants or defects. The TRPL decay curves are fitted with a bi-exponential dependence, where the quick process (short carrier lifetime) is brought by bimolecular recombination at the beginning stage and the slow decay process (long carrier lifetime) is brought by monomolecular recombination at a long-time scale. For perovskites, bimolecular recombination is caused by the recombination of photogenerated electrons and holes, while monomolecular recombination is mainly caused by SRH recombination. According to the decay curve, bimolecular and monomolecular recombination first decreases and then increases with an increasing precursor ratio. In the perovskite film with a PbI_2_/MAI ratio of 0.9, both bimolecular and monomolecular recombination rates are high. The samples at the PbI_2_/MAI ratio of 1.0 exhibit the lowest bimolecular and monomolecular recombination, indicating a lower defect density. Moreover, the absorption spectra of perovskite films (Glass/ITO/MAPbI_3_) were measured to analyze the absorbance of perovskite with different precursor ratios, as shown in Figure S1, Supplementary Materials.

**Figure 7:**
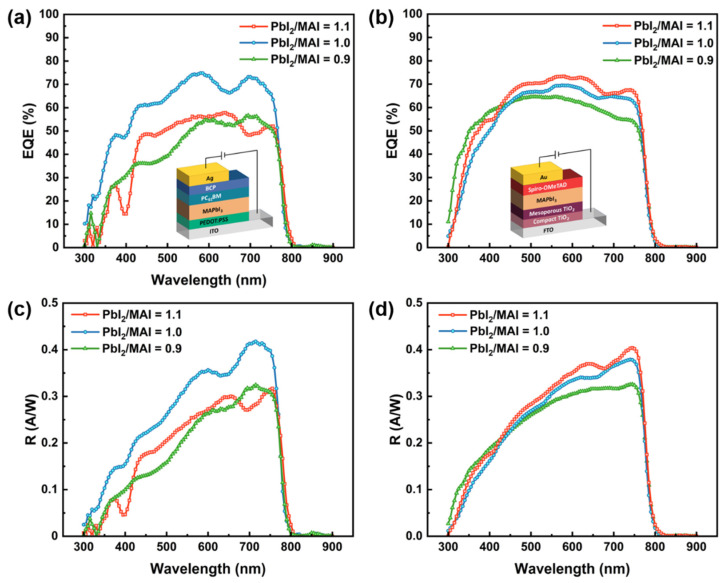
Variation in EQE and responsivity with wavelength under different precursor ratios annealed at 80 °C for 10 min, where (**a**,**c**) are for inverted structure photodiode and (**b**,**d**) are for regular structure photodiode.

The EQE spectra of devices fabricated with different precursor ratios under the condition of annealing at 80 °C for 10 min in self-powered mode are shown in Figure 7a,b. Except for the precursor ratio variation, the specific preparation and characterization of the inverted and regular perovskite photodiodes can be referred to in our previous work [19]. Attributed to high-quality perovskite films, EQEs can still be obtained by changing the precursor ratios with fixed annealing conditions. The optimal performance of inverted and regular structure photodiodes is achieved at precursor ratios of 1.0 and 1.1, respectively, where the maximum EQE of the inverted structure is 74.83% at 580 nm, whereas the highest EQE of the regular structure is 73.36% at 580 nm. According to Figure 7c,d, the highest responsivity of inverted and regular structures is 0.417 and 0.404 A/W, respectively. Combined with the analysis in the above section, changing the precursor ratio can adjust the doping type and density of perovskite. The inverted structure is lightly P-doped with the maximum EQE when the precursor ratio is 1.0, while the regular structure reaches the maximum EQE with N-type doping at a precursor ratio of 1.1. Based on the optimal doping condition of perovskite films at the precursor ratio of 1.0, the dark currents of inverted-structure perovskite photodiode were measured with applied voltage ranging from −1 to 1 V, which are exhibited in Figure S3, Supplementary Materials. For high-sensitivity perovskite photodiodes, dark current is also a key parameter affecting perovskite PD stability, which is mainly caused by the leakage current of trapped charge carriers or bulk charge injection under reverse bias [30]. Notably, the dark current of the photodiode is still restrained by the built-in electric field despite light doping at the precursor ratio of 1.0. The lowest dark current of the inverted structure photodiode reaches 1.03 × 10^−9^ A in the self-powered mode.

## 3. Methods

In order to describe the generation process of photogenerated carriers, the generation rate is used to express the number of electron-hole pairs generated along the surface normal direction of the device. Because the carrier distribution within the device surface plane is axisymmetric, only the carrier distribution along the surface normal direction needs to be considered allowing for one-dimensional (1D) simplification. Consequently, the generation of photocarriers at each position of the device surface normal direction can be calculated according to Equation (3), where α is the absorption coefficient; x is the distance from the light incident surface to the calculated given position in the material, where the light incident surface of the material is the origin coordinate of the position (material is PEDOT: PSS for inverted structure and compact TiO_2_ for regular structure); $N_{0}$ refers to the photon flux at the glass substrate/air interface, which is defined as the number of photons per unit area per second [31]. It is also noteworthy that in the commercial software of Setfos or GPVDM the light absorption calculation also considers the interferences and transfer matrix formalism are adopted. However, Equation (1) is still the choice of much of the published work and is more easily understood as the starting point to analyze the physical process.

(3) $${G=\alpha N}_{0}\cdot \exp(-\alpha x)$$

The perovskite films possess slow decay caused by trap-assisted recombination, which is also called the Shockley-Read-Hall (SRH) recombination as shown in Equation (4). The SRH model of the carrier trap recombination is applicable for the regions with impurity conductivity [32]:

(4) $$R_{SRH}=C_{t}N_{t}\frac{np-n_{i}^{2}}{\left(n+n_{t})+(p+p_{t}\right)}$$

(5) $$C_{t}=\mu \cdot \frac{\left|V_{app}-V_{bi}\right|}{\delta }\cdot \frac{5}{N_{t}^{2/3}}$$

(6) $$n_{t}=N_{c}\cdot \exp(\frac{E_{t}-E_{c}}{kT})$$

(7) $$p_{t}=N_{v}\cdot \exp(\frac{E_{v}-E_{t}}{kT})$$

where $N_{t}$ is the trap density; $C_{t}$ is the capture rate of traps, which is calculated by Equation (5), where μ is the mobility, δ is the layer thickness, $V_{app}$ is the applied voltage, and $V_{bi}$ refers to the built-in potential; $n_{t},p_{t}$ refer to trapped electron and hole densities, which is given by Equations (6) and (7); $N_{c},N_{v}$ are the effective densities of states in the conduction band (CB) and valence band (VB); $E_{c},E_{v}$ are the energy levels of the CB bottom and VB top; $E_{t}$ is the trap energy level; *k* is the Boltzmann constant; and *T* is the temperature of the semiconductor.

The built-in electric fields *E*s are formed at the heterojunction interfaces between the active and the transport layers due to the difference in energy levels for both the inverted and regular structure photodiodes [33,34]. Moreover, the strength and direction of the interfacial electric field can be changed by controlling the perovskite film doping type and density. The *E* can be calculated from Poisson’s equation [35,36]. As mentioned above, the device can be viewed as being in 1D with doping and traps, and the *E* is expressed as follows:

(8) $$\frac{dE}{dx}=\frac{q}{\varepsilon \varepsilon_{0}}(p-n+p_{t}-n_{t}-N_{A}+N_{D})$$

where $N_{A}$ (Acceptor) and $N_{D}$ (Donor) is the doping term; n,p are the free electron and hole densities; $n_{t},p_{t}$ are trapped charges; q is the elementary charge; and ε represents the dielectric constant of active layer materials.

Under the influence of the built-in *E*s, the carriers are separated, and the interfacial *E* promotes the drift of holes to the HTL and the electrons to the ETL, eventually reaching dynamic equilibrium. Based on the drift-diffusion model of charge carriers, current densities distribution caused by the drift-diffusion of holes and electrons can be computed by Equations (9) and (12) [37]:

(9) $$\vec{J_{n}}=q\mu_{n}n\vec{E}+qD_{n}\vec{\nabla }n$$

(10) $$\vec{J_{p}}=q\mu_{p}p\vec{E}-qD_{p}\vec{\nabla }p$$

(11) $$D_{n}=\mu_{n}\frac{kT}{q}$$

(12) $$D_{p}=\mu_{p}\frac{kT}{q}$$

where the currents are composed of a drift and a diffusion term; $D_{n},D_{p}$ are the diffusion constants; and $\mu_{n},\mu_{p}$ are the electron and hole mobilities.

During the transient process of electron-hole pairs generation, electrons and holes are equal in number. After carrier recombination, drift, and diffusion, the number of electrons and holes eventually reaches steady equilibrium. Two boundary conditions are formed when the carriers reach a steady state, namely, $\frac{dn}{dt}=\frac{dp}{dt}=0$. Therefore, based on the above description of carrier generation and recombination, the carrier density can be calculated from the continuity Equations (13) and (14):

(13) $$\frac{dn}{dt}=\frac{\vec{\nabla }\vec{J_{n}}}{-q}-R_{SRH}+g_{opt}G$$

(14) $$\frac{dp}{dt}=\frac{\vec{\nabla }\vec{J_{p}}}{q}-R_{SRH}+g_{opt}G$$

where $\vec{J_{n}},\vec{J_{p}}$ are the current densities given above; $R_{SRH},G$ are the recombination and generation rates of electron-hole pairs; and $g_{opt}$ is the generation efficiency.

In addition, there are several key parameters used to quantitatively evaluate the performance of perovskite photodiodes, including the external quantum efficiency (EQE), responsivity (*R*), and detectivity (D*). The EQE is the ratio of the output photocurrent to the incident photon current. The EQE can be calculated by Equation (15), where $J_{ph}$ is the photocurrent density, h is Planck’s constant, c is the speed of light, and λ is the light wavelength. The light intensity I is given by Equation (16), where $I_{0}$ is the light intensity at the light incident surface of the devices. Responsivity is defined as the ratio of photocurrent to the incident light power on the active area of photodiodes as shown in Equation (17) [38,39,40].

(15) $$\mathrm{EQE}=J_{ph}/\int_{\lambda }\frac{q\lambda I(\lambda )d\lambda }{hc}$$

(16) $$I(\lambda )=I_{0}(\lambda )\cdot \exp(-\alpha x)$$

(17) $$R=\mathrm{EQE}\cdot \frac{\lambda }{hc}$$

In this work, multiple characterization techniques were adopted and the conditions are briefly summarized. A scanning electron microscope (SEM) was used to characterize the surface morphologies of the as-prepared MAPbI_3_ films and the cross-sectional morphology of the PDs (ZEISS Gemini-300). By using X-ray diffraction ([XRD] Rigaku SmartLab 9 kW system, Cu-K α radiation λ = 0.154 nm), the chemical compositions and structures of the perovskite films were analyzed and the samples were scanned from 10° < 2θ < 40°. X-ray photoelectron spectroscopy ([XPS] Thermo K-α+) was employed to measure the perovskite films’ valence and XPS spectra. All spectra were shifted using inorganic carbon at 284.80 eV as a reference to take sample charging into consideration. Time-resolved photoluminescence (TRPL) decay was measured with a FluoTime 300 under 532 nm pulse laser irradiation. A UV/Vis/NIR spectrophotometer (PerkinElmer Lambda 950) was used to measure the absorption spectra of the perovskite films.

## 4. Conclusions

In this study, a coupled opto-electronic model is constructed to systematically analyze the influence of factors on the performance of doped p-i-n perovskite photodiodes with both regular and inverted structures. The operational mechanism of doped perovskite photodiodes and the physical conversion of light to electricity are revealed by numerical calculation and experimental verification of the doping type and density variation using a precursor ratio variation strategy. The generation rate of photogenerated carriers is initially calculated followed by carrier density distribution, which is the core joint part of the coupled opto-electronic model whereby the electric field strength, recombination rate, and current density of carriers are further analyzed. Within the coupled opto-electrical model, the strength and direction of the built-in electric field are analyzed by the doping type and density of the perovskite. Based on the optimal conditions, which included annealing at 80 °C for 10 min and setting the precursor ratio to 1.0 for inverted structure and 1.1 for regular structure, the inverted and regular perovskite photodiodes obtained an EQE of 74.83% and 73.36% and responsivity of 0.417 and 0.404 A/W, respectively. Both the coupled opto-electronic model and experimental characterizations confirm the positive effect of the built-in electric field on improving the performance of perovskite photodiodes. The coupled opto-electronic model and analysis method proposed in this study provide significant guidance for the future design and performance enhancement of perovskite photodiodes.

## Data Availability

Not applicable.

## Supplementary Materials

The following supporting information can be downloaded at: https://www.mdpi.com/article/10.3390/molecules27196223/s1. Table S1: The parameters of regular structure, Table S2: The parameters of inverted structure, Table S3: Element ratio by XPS with different precursor ratios, Figure S1: Normalized absorption spectra of perovskite thin films with different precursor ratios, Note S1: Absorption test results of perovskite films, Figure S2: Simulated built-in electric field for various perovskite doping type and densities, Figure S3: Dark current-voltage characteristic curve of inverted structure annealed at 80 °C for 10 min with a 1.0 precursor ratio.

## Sample Availability

Samples of the compounds are not available from the authors.
