# Supplementary material for: Revealing the Hidden Mechanism of Enhanced Responsivity of Doped p-i-n Perovskite Photodiodes via Coupled Opto-Electronic Model
Source: Molecules. 2022 Sep 22;27(19):6223. doi: 10.3390/molecules27196223 (PMC9571005; doi:10.3390/molecules27196223)
Supplement: Supplementary file 1 [file molecules-27-06223-s001.zip › molecules-1900445-supplementary.pdf]

molecules

Revealing the Hidden Mechanism of Enhanced Responsivity of Doped p-i-n Perovskite Photodiodes via Coupled Opto-Electronic Model

Dan Wu [#*1], Hechun Zhang [#1], Haochen Liu [2], Wenhui Li [2], Xiangtian Xiao [2], Kanming Shi [2], Taikang Ye [2], Jiayun Sun [2], Zhaowen Lin [1], Jing Liu [1], Mingxia Qiu [1], Aung Ko Ko Kyaw [2], and Kai Wang [2]

[1]College of New Materials and New Energies, Shenzhen Technology University, Shenzhen, 518118, People's Republic of China

[2]Department of Electrical and Electronic Engineering, Southern University of Science and Technology, Shenzhen, 518055, People's Republic of China

*Correspondence: wudan@sztu.edu.cn

[#]These authors contribute equally

Supplementary Materials: Table S1: The parameters of regular structure, Table S2: The parameters of inverted structure, Table S3: Element ratio by XPS with different precursor ratios, Figure S1: Normalized absorption spectra of perovskite thin films with different precursor ratios, Note S1: Absorption spectrum of perovskite films, Figure S2: Simulated built-in electric field for various perovskite doping type and densities, Figure S3: Dark current-voltage characteristic curve of inverted structure annealed at 80 ℃ for 10 min with 1.0 precursor ratio.

Table S1. The parameters used for evaluation of the built-in electric fields for inverted structure perovskite photodiode.

	**PEDOT: PSS**	**$MAPbI_3$**	**$PC_{61}BM$**	**BCP**
Thickness(nm)	10	360	90	5
LUMO (eV)	-3.6	-3.725	-4.3	-3
HOMO (eV)	-5.0	-5.325	-5.3	-6.5
Dielectric constant	3.5	25	3.5	3
N_0(cm^{-3})	1E21	2.5E18	1E20	1E20
Acceptor doping (cm^{-3})	1E20	/	/	/
Donor doping (cm^{-3})	/	/	1E17	1E19
Electrons mobility(cm^2/Vs)	8E-4	10	0.001	1E-6
Holes mobility(cm^2/Vs)	8E-4	10	0.001	1E-6

Table S2. The parameters used for evaluation of the built-in electric fields for regular structure perovskite photodiode.

	**Compact TiO_2**	**Mesoporous TiO_2**	**$MAPbI_3$**	**Spiro-OMeTAD**
Thickness(nm)	40	160	360	160
LUMO (eV)	-4.1	-4.1	-3.725	-2.9
HOMO (eV)	-7.2	-7.2	-5.325	-5.22
Dielectric constant	100	100	25	3

N_0(cm^{-3})	4.5E20	4.5E20	2.5E18	1E18
Acceptor doping (cm^{-3})	/	/	/	1E19
Donor doping (cm^{-3})	6.2E18	6.2E18	/	/
Electrons mobility(cm^2/Vs)	0.006	0.006	10	0.001
Holes mobility(cm^2/Vs)	0.006	0.006	10	0.001

Table S3. Element ratio of the lead and iodine by X-ray photoelectron spectroscopy (XPS) with different precursor ratios.

**Precursor ratio**	**Pb**	**I**	**I/Pb ratio**
PbI_2/MAI=0.9	11.35%	39.13%	3.45
PbI_2/MAI=1.0	12.68%	40.76%	3.21
PbI_2/MAI=1.1	15.82%	43.02%	2.72

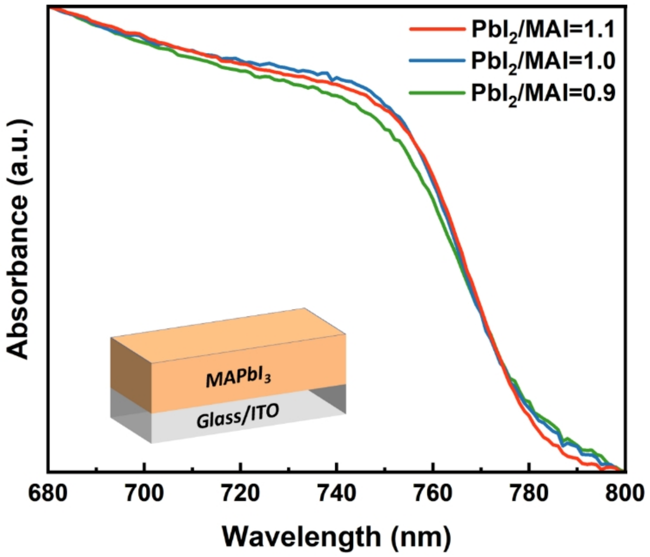

Figure S1. Normalized absorption spectra of perovskite thin films (Glass/ITO/$MAPbI_3$) with different precursor ratios.

Note S1. Absorption test results of perovskite films (Glass/ITO/$MAPbI_3$).

As shown in Figure S1, increasing the ratio of precursors reduces the absorbance of perovskite thin films at long wavelengths. It can be observed that the spectrum appears red-shifted with the increase of the precursor ratio by Figure S2, indicating that the photon capture ability is greater when the precursor ratio is 1.0 and 1.1 than when the precursor ratio is 0.9. By adjusting the appropriate precursor ratio, the perovskite film can enhance the photon capture ability and high crystallinity, which is beneficial for the preparation of devices with high responsivity from the ultraviolet band to the near-infrared band.

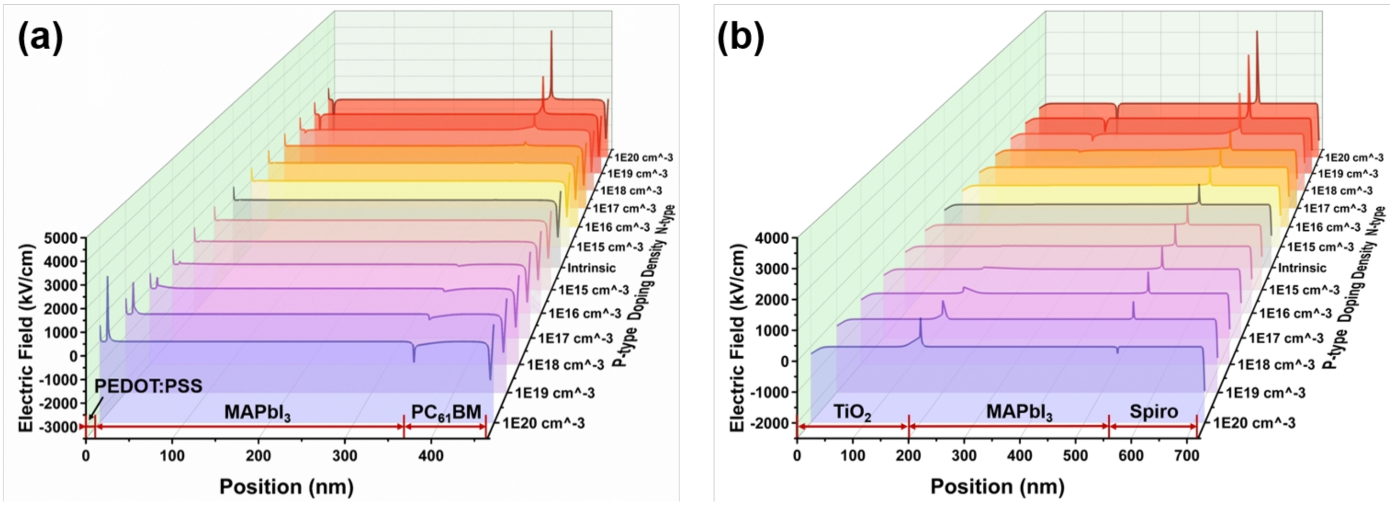

Figure S2. Simulated built-in electric field for various perovskite doping type and densities of the (**a**) inverted and (**b**) regular structures.

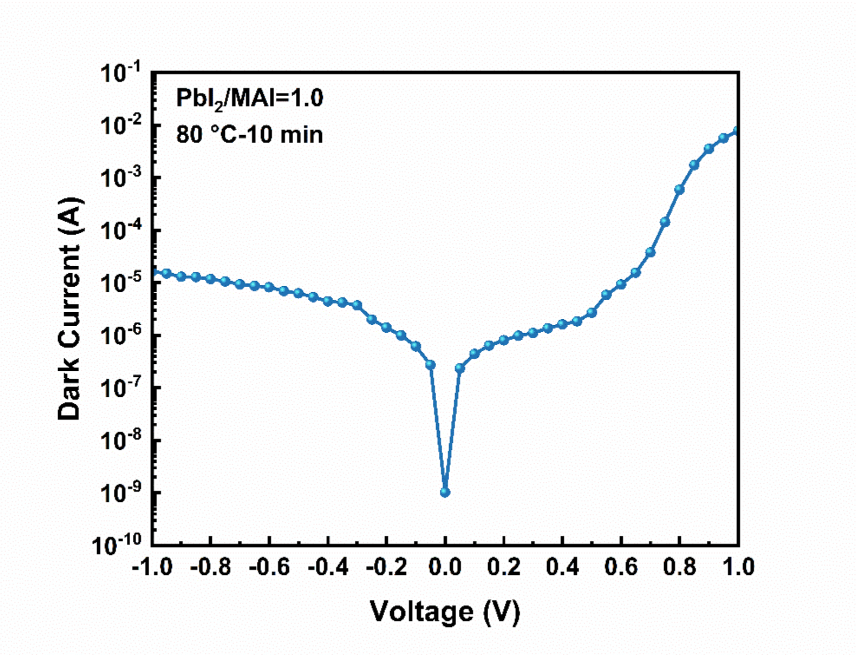

Figure S3. Dark current-voltage characteristic curve of inverted structure annealed at 80 ℃ for 10min with 1.0 precursor ratio.
